# Genome-Wide Identification and Functional Characterization of the Heat Shock Factor Family in Eggplant (*Solanum melongena* L.) under Abiotic Stress Conditions

**DOI:** 10.3390/plants9070915

**Published:** 2020-07-20

**Authors:** Jinglei Wang, Haijiao Hu, Wuhong Wang, Qingzhen Wei, Tianhua Hu, Chonglai Bao

**Affiliations:** Institute of Vegetables Research, Zhejiang Academy of Agricultural Sciences, Hangzhou 310021, China; syauwjl@163.com (J.W.); huhj0571@126.com (H.H.); hongge5@163.com (W.W.); weiqz@mail.zaas.ac.cn (Q.W.); hutianh@126.com (T.H.)

**Keywords:** eggplant, heat shock factor, gene family, expression profile, abiotic stress

## Abstract

Plant heat shock factors (Hsfs) play crucial roles in various environmental stress responses. Eggplant (*Solanum melongena* L.) is an agronomically important and thermophilic vegetable grown worldwide. Although the functions of Hsfs under environmental stress conditions have been characterized in the model plant *Arabidopsis thaliana* and tomato, their roles in responding to various stresses remain unclear in eggplant. Therefore, we characterized the eggplant SmeHsf family and surveyed expression profiles mediated by the SmeHsfs under various stress conditions. Here, using reported Hsfs from other species as queries to search SmeHsfs in the eggplant genome and confirming the typical conserved domains, we identified 20 SmeHsf genes. The SmeHsfs were further classified into 14 subgroups on the basis of their structure. Additionally, quantitative real-time PCR revealed that SmeHsfs responded to four stresses—cold, heat, salinity and drought—which indicated that SmeHsfs play crucial roles in improving tolerance to various abiotic stresses. The expression pattern of *SmeHsfA6b* exhibited the most immediate response to the various environmental stresses, except drought. The genome-wide identification and abiotic stress-responsive expression pattern analysis provide clues for further analysis of the roles and regulatory mechanism of SmeHsfs under environmental stresses.

## 1. Introduction

Plants have developed various defense mechanisms that are responsive to different environmental stresses, such as drought, cold, salinity, and heat [1]. Transcription factors, like AP2/ERF, HSP90, WRKY, MYB, NAC, LOX, bZip, and heat shock (Hsfs) [2,3,4,5,6,7,8], are activated and regulate multiple genes and signaling pathways that enable plant adaptation to unfavorable conditions. Among them, Hsfs are involved in many aspects of protein homeostasis under stress conditions [9] and are especially involved in responding to high-temperature stress [10]. In addition to stress responses, Hsfs also play important roles in developmental processes in animals and plants [11].

Although the sequences and sizes of *Hsf* genes vary, the basic structures and promoter recognition modes are considerably conserved in higher eukaryotes [12]. Almost all the Hsfs have a highly conserved DNA-binding domain (DBD), located close to the N-terminus and containing an antiparallel four-stranded β-sheet and a three-helical bundle, which are required for specific binding with heat stress promoter elements [13,14,15]. The oligomerization domain (HR-A/B region), separated from the DBD domain by a flexible linker of a variable length, contributes to the trimerization of Hsfs by forming a coiled-coil structure [16]. Additionally, three other conserved structures—a nuclear localization signal (NLS), nuclear export signal (NES) and activator motif (AHA)—are present. Some Hsfs also contain a repression domain at the C-terminus [17]. On the basis of structural characteristics and phylogenetic comparisons, plant *Hsf* genes can be further divided into A, B, and C classes [12,18], which contain insertions of 21, 0, and 7 amino acid residues, respectively, between the HR-A and HR-B regions [12,18]. In addition, the amino acid length from the DBD to HR-A/B differs among the three classes [12]. The AHA is present in class A, but absent in classes B and C [17]. Class A Hsfs are involved in transcriptional activation and responses to environmental stresses [19], while class B Hsfs function as transcriptional coactivators with class A Hsfs or as gene expression repressors [9,20]. At present, there are few studies on class C; only several studies show that class C Hsfs can be induced by a variety of stresses [21,22].

Hsfs are engaged in responses to abiotic stresses conditions. For example, *Arabidopsis thaliana HSFA1*s and *HSFA2* participate in responses to various abiotic stresses, such as salinity, osmotic pressure, oxidation, and anoxia [23,24,25], while *HSFA1b* and *HSFA*3 are involved in drought-stress responses [17,26]. Tomato *HsfA1a* plays a critical role in the development of thermotolerance and cannot be replaced by other tomato *Hsf*s [27]; *HsfA1b* and *HsfA1e* are likely responding to stress in specific tissues, while *HsfA1c* functions as a co-regulator in mild heat stress response. Tomato *HsfA2* can increase plant heat tolerance by accumulating to high levels [28] and is also involved in protecting maturing and germinating pollen under heat-stress conditions [29]. In addition, wheat *HsfA4a* is involved in cadmium tolerance [19]. Chrysanthemum *HSFA4* confers salinity tolerance as a consequence of Na^+^/K^+^ ion and reactive oxygen species homeostasis [30]. *HSFA2* and *A6* from wheat, *HSF3*, *-18*, *-24*, *-32*, *-37*, and *-40* from cotton and *HSF-06*, *-10*, *-14*, *-20*, and *-21* from maize may be involved in responding to heat stress [22,31,32]. Owing to their essential modulatory functions in plants, *Hsf* gene family members have been studied in several agronomically important plants, such as rice (*Oryza sativa*), maize (*Zea mays*), apple (*Malus domestica*), poplar (*Populus trichocarpa*), and cabbage (*Brassica oleracea*) [31,33,34,35,36]. However, the *Hsf* gene family in eggplant (*Solanum melongena* L.) has not been systematically studied.

Eggplant is an economically important vegetable cultivated worldwide. The optimal season for eggplant growth is autumn, when the temperature ranges from 22 to 30 °C [37]. During year-round production in protected cultivation, eggplant encounters various environmental stresses, including heat, cold, drought, and salinity. Here, we performed a genome-wide study to comprehensively analyze the eggplant *Hsf* gene family. We identified 20 *SmeHsf* genes and determined protein properties, phylogenetic relationships, gene structures, and conserved protein domains. We also investigated the expression changes of *Hsf* genes in plants subjected to different abiotic stresses. Our study provides a foundation for further *SmeHsf*s functional investigations and could help better understand the environmental stress-response-related molecular mechanisms of *Hsf* genes in eggplant. 

## 2. Results

### 2.1. Identification, Classification, and Characterization of the Hsf Gene Family in Eggplant

A total of 20 *Hsf* genes were identified in the eggplant genome (Table S1). This is less than in pepper (25), tomato (26), potato (25), and cultivated tobacco (65). Subsequently, the 20 *SmeHsf* genes were classified into three subgroups—A, B, and C—according to the HEATSTER websites [38]. Most of the SmeHsfs, 14 out of 20, were classified into subgroup A, and these SmeHsfs were further classified into seven subgroups (A1, A3, A4, A5, A6, A8, and A9). Class B had five members from four subgroups (B1, B2, B3, and B4). Subgroups A1, A4, A6, A9, and B2 contained more than one member (Table 1).

The physical and chemical properties of the SmeHsf proteins were analyzed and some differences were observed. The lengths of SmeHsf proteins varied from 213 to 496 amino acids, and the molecular weights ranged from 24.62 to 55.07 kDa. Among the 20 SmeHsf proteins, SmeHsfB3a had the shortest length and lowest molecular weight. Additionally, the predicted aromaticity ranged from 0.05 to 0.11, and the isoelectric point ranged from 4.60 to 9.44 (Table 1). The instability index, which provides an estimate of the stability of a protein in a test tube, indicated that all the SmeHsf proteins are unstable (scores greater than 40), except SmeHsfB1, which had an instability index of 30.35. The grand average of hydropathy values of the SmeHsfs was less than 0, suggesting that they are hydrophilic. These differences mostly resulted from variations in the non-conserved regions’ amino acid sequences.

### 2.2. Conserved Domains and Structural Analysis of SmeHsfs

**Figure 1:**
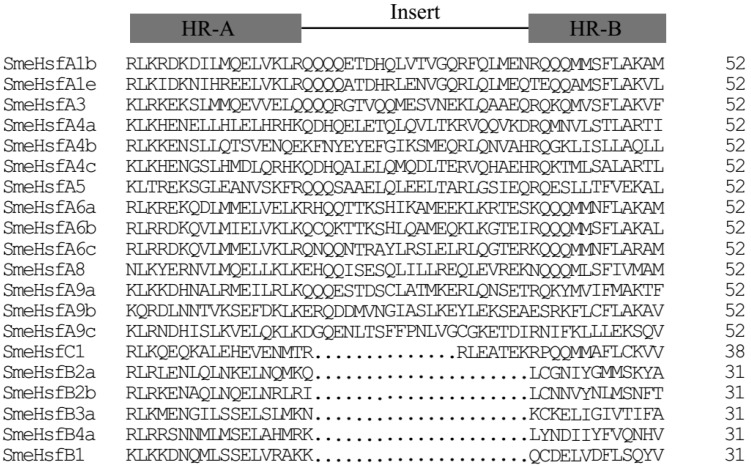
Multiple sequence alignment of the HR-A/B regions (OD) of SmeHsfs.

The functional domains of the Hsfs have been studied in some model plants [11]. Detailed information regarding the conserved domains, such as DBD, HR-A/B, NLS, NES, and AHA, are presented in Table 2. As the core functional domain of the Hsfs, the DBD was composed of approximately 90 amino acids and existed in all the predicted SmeHsf proteins. In addition, another conserved domain, HR-A/B, was also present in all the SmeHsfs. Based on the number of amino acid residues inserted into the HR-A/B regions, the 20 SmeHsfs were divided into three major classes. Class A and class C Hsfs contained 21 and 7 amino acid residues between the A and B regions, respectively (Figure 1). This classification confirmed the results of HEATSTER website. Most of the SmeHsf proteins (13 out of 20) included an NLS domain, and the NES domain was detected in seven SmeHsfs. The AHA domain was detected in the A4, A5, A6, and A9 subgroups; however, it was not found in class B or C.

**Figure 2:**
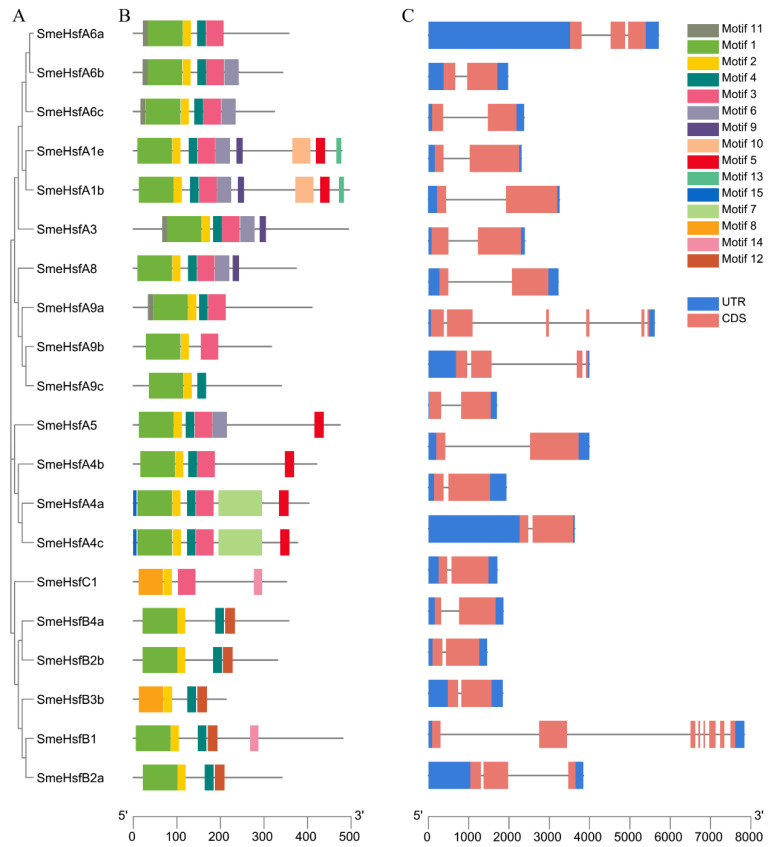
Phylogenetic, motif and structural analyses of SmeHsfs. (**A**) Phylogenetic tree of SmeHsf proteins. (**B**) Schematic representation of the motif compositions of SmeHsfs. (**C**) Exon/intron structures of *SmeHsf* genes.

To further analyze the motifs and structural variations of SmeHsfs, we constructed a separate phylogenetic tree containing only SmeHsf proteins, and then, compared motif compositions and exon/intron organizations (Figure 2A). Generally, most of the closely related members had similar motif compositions and exon/intron organizations and lengths. The Multiple Em for Motif Elicitation (MEME) web server was used to search for motifs in the SmeHsf proteins. There were 15 potential motifs distributed throughout the Hsf protein sequences (Figure 2B). Motifs 1 and 2, or Motifs 2 and 8, which corresponded to the DBD domain, were found in all the SmeHsfs. Motif 4 was also identified in all the SmeHsfs and corresponded to the HR-A/B region. Different subgroups had similar motifs and contained their own unique motifs. Motif 3 was found in class A and C members, while Motif 12 was only found in class B members.

The exon/intron structures exhibited a highly conserved organization in 14 out of 20 *SmeHsf*s possessing strictly two exons, which was similar to the structures of *Hsf* genes in other plants [39,40]. In addition, we identified two genes possessing three exons (*SmeHsfA6a* and *SmeHsfB2a*), one gene containing four exons (*SmeHsfA9b*), and two genes (*SmeHsfA9a* and *SmeHsfB1*) having more than six exons (Figure 2C).

### 2.3. Phylogenetic Analysis of SmeHsfs

**Figure 3:**
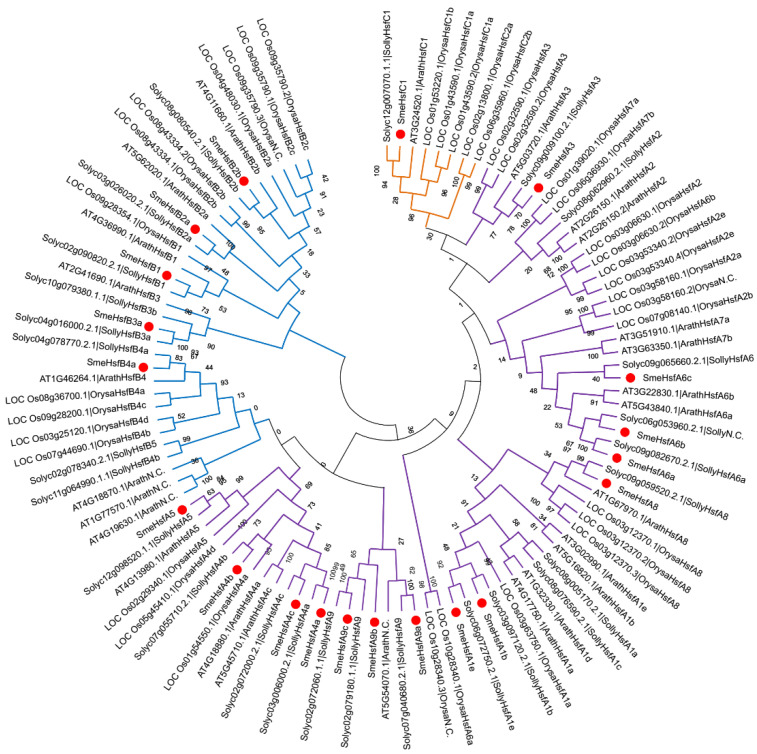
The phylogenetic tree of the *Hsf* genes from four plant species. Individual species are distinguished by different gene code prefixes. The prefixes Arath, Orysa, Solyc, and Sme indicate that these genes are from *A. thaliana*, rice, tomato, and eggplant, respectively. Red circles indicate eggplant genes. Additionally, purple, blue, and yellow branches indicate classes A, B, and C, respectively.

To study the evolutionary characteristics of SmeHsf proteins, we selected three other well-studied and representative plant species, including one related species (*Solanum lycopersicum*), a monocot (*O. sativa*), and a eudicot (*A. thaliana*). The full-length amino acid sequences of Hsf proteins in eggplant and these three species were used to construct a phylogenetic tree (Figure 3 and Table S2). The *SmeHsf*s in the same subgroup were classified together, which indicated that the *SmeHsf*s in the same subgroup not only have similar domain structures, and also, have similar sequences. The phylogenetic analysis also showed that the number of *Hsf* genes in different subclasses varied among land plants. For example, eggplant has no subclass A2 members, while rice has no subclass A9 and B3 members. Besides, the *Hsf* genes only varied slightly in different subclasses between eggplant and tomato, indicating an even distribution within the family Solanaceae.

### 2.4. Putative Regulatory cis-Elements of the SmeHsf Promoters

**Figure 4:**
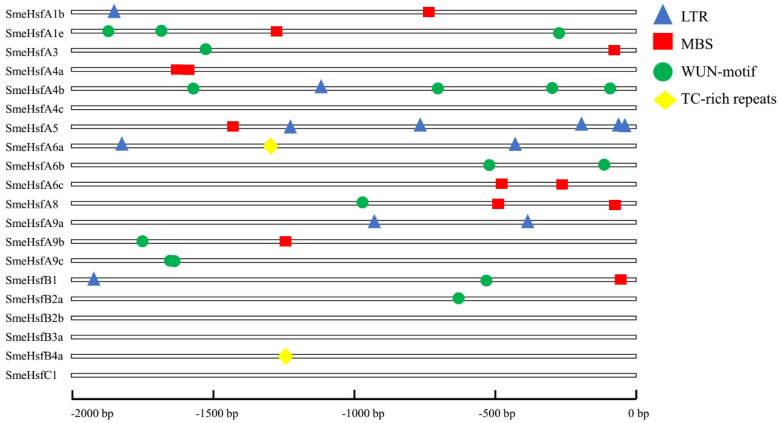
The position of abiotic stress-response *cis*-elements on *SmeHsf* promoters.

To further explore the potential regulatory mechanisms of *SmeHsf*s during stress responses, we used the PlantCARE database [41] to detect the *cis*-elements in the promoters (Table S3). In total, 88 *cis*-elements were identified, with 55 having known functions. The most commonly known function was responsiveness to light (26 out of 55), followed by other regulatory functions (14 out of 55), and responsiveness to hormones (9 out of 55). In addition, four abiotic stress-response elements—LTRs, MBSs, TC-rich repeats, and WUN motifs—were identified. The SmeHsfs, except for *SmeHsfA4c*, *SmeHsfB2b*, *SmeHsfB3a*, and *SmeHsfC1*, possessed at least one stress-response-related *cis*-element (Figure 4). In total, six *SmeHsf*s had one or more LTR, suggesting a potential cold-stress response under low temperature conditions. Additionally, MBSs, TC-rich repeats, and WUN motifs were found in 9, 2, and 10 *SmeHsf*s, respectively. The *cis*-element analysis indicated that *SmeHsf* genes could respond to different abiotic stresses.

### 2.5. qRT-PCR Analysis of SmeHsf Responses to Different Abiotic Stresses 

**Figure 5:**
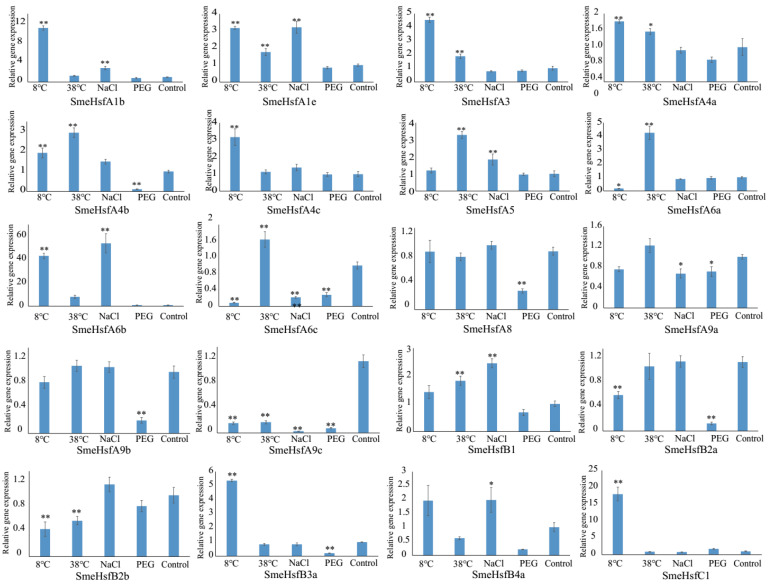
qRT-PCR analysis of *SmeHsf* genes under various abiotic stress conditions. The expression level of the control CK treatment was normalized as 1.0. The results are shown as means ± SDs of three independent experiments. The significant differences at *p* ≤ 0.05 and *p* ≤ 0.01 are represented by one and two asterisks, respectively.

The expression levels of *Hsf* genes are affected by heat and other abiotic stresses in plants [42]. In this study, we analyzed the expression levels of *SmeHsf*s under different stress conditions, including cold, heat, salinity, and drought, to determine the stress-responsive candidates (Figure 5). The expression level of *SmeHsfA6b* dramatically increased 43-, 54-, and 8-fold under cold, salinity, and heat treatments, respectively, indicating its function in increasing plant adaptability to these abiotic stresses. In total, 14, 10, 9, and 8 *SmeHsf*s showed significant differential expression levels under cold, heat, salinity, and drought treatments, respectively. Thus, the functions of these stress-induced *SmeHsf*s should be analyzed in further studies. Overall, the average ranges of expression level changes of these *SmeHsf*s under cold conditions were greater than those identified under other stress conditions. Under cold-stress conditions, the expression levels of *SmeHsfC1* and *SmeHsfA1b* increased more than 10-fold, while those of *SmeHsfA3*, *SmeHsfA4c*, and *SmeHsfB3a* increased 3–5-fold. In addition, the expression levels of *SmeHsfA5* and *SmeHsfA6a* were upregulated approximately 3–4-fold in response to a heat treatment.

## 3. Discussion

Eggplant is an important vegetable belonging to the Solanaceae family, which encompasses crops like tobacco, tomato, potato, and pepper. The Hsfs act as terminal components of signal networks that participate in various abiotic stress responses [43]. Hsfs can regulate the expression of molecular chaperones, such as heat shock proteins, which are involved in heat-stress responses [44] and regulate the signaling networks of stress-related phytohormones, such as salicylic and abscisic acids [32,45]. However, a comprehensive characterization of the *Hsf* gene family in eggplant is lacking. 

In this study, we identified 20 *SmeHsf* genes. In Solanaceae species, the genome sizes and gene numbers of eggplant (833.1 Mb and 42,035 coding genes, respectively), potato (844 Mb and 35,119 coding genes), and tomato (950 Mb and 34,727 coding genes) are similar [46,47,48,49]; however, eggplant has the lowest number of *Hsf* genes. Notably, the genome size of pepper (3.48 Gb) was approximately fourfold larger than those of these three species, but the coding genes (34,899) and *Hsf* gene numbers did not vary significantly [49]. However, cultivated tobacco, which has an almost fivefold larger genome size (4.41–4.57 Gb) than that of eggplant and has a high number of coding genes (85,439 coding genes) [50], has more than twice the number of *Hsf* genes than eggplant. Thus, the number of *Hsf* genes is not correlated with the genome size, but is proportionally related to the total number of coding genes. Consequently, because pepper is a diploid species and contains a large number of repetitive sequences [49], it has less *Hsf* genes compared with the tetraploid cultivated tobacco, which has undergone an allopolyploidization event.

An unrooted phylogenetic tree was constructed using previously reported Hsfs and SmeHsfs. The SmeHsfs in the same subgroups clustered together, corresponding to other *Hsf* genes, which confirmed the SmeHsf classification. Class A was the predominant class in both monocots and dicots. Like the Hsfs in other plants, all 20 SmeHsfs contained conserved DBD and HR-A/B domains, which are essential for their transcriptional functions. Although the overall gene structure of SmeHsfs in the A, B, and C classes were similar, the different groups contained characteristic domains. 

Expression profile changes of Hsf genes that occur under various abiotic stresses have been extensively analyzed in different plants [40,51,52,53]. Investigating the expression changes of *SmeHsf*s under different stresses provides clues to their functions. *SmeHsf*s responded to the four stresses including heat, cold, salinity, and drought, which indicated that *SmeHsf*s increase tolerance levels to various abiotic stresses. Up to the present, many researchers found that class A and B Hsfs are involved in responding to environmental stresses, and few focused on the class C. In our study, we found that all class A and B SmeHsfs up- or downregulated under at least one stress, which indicated their functions in responding to environmental stresses. Interestingly, the expression level change of *SmeHsfC1* was only less than that of *SmeHsfA6b* under low temperature treatment, which indicated the class C member also plays a role in responding to low temperature stress in eggplant.

*Hsf* genes are expected to always respond to heat stress [54]. However, more *SmeHsf*s showed significant differential expression levels and greater ranges in expression changes under cold conditions than under other stress conditions, which might be because eggplant is a warm-weather plant that is more sensitive to low temperature [55]. More *SmeHsf*s showed significant differential expression levels under cold- and heat-stress conditions than under saline and drought conditions, which might be because leaf tissue was detected in this study and the leaves being the first organs to perceive heat and cold stresses, while the roots are the first organs to sense drought and salinity stresses [40]. *AtHsfA6a* and *AtHsfA6b* participate in abscisic acid-mediated thermotolerance and drought tolerance [32]; however, the wheat *HsfA6*, which is the most inducible wheat *Hsf* gene, is only responsive to the oxidative stress-signaling pathway [40]. In our study, *SmeHsfA6b* was also the most inducible *SmeHsf* gene, being upregulated by cold, heat and salinity treatments in the leaves, but not in response to the drought stress, which indicated that homologous *Hsf* genes have different functions in different plants. Moreover, in tomato, *HsfA1* plays a leading role in the heat-shock reaction and combines with *HsfA2* to form a complex that increases plant heat tolerance [28]. However, *HsfA2* was not identified in eggplant. Thus, the expression analysis indicated that *SmeHsf*s respond in unique manners to various environmental stresses, and the responses of these *SmeHsf*s are different in both magnitude and sensitivity to the above stresses.

## 4. Materials and Methods 

### 4.1. Identification and Characterization of Hsfs in Eggplant

The eggplant genome (version SME_r2.5.1) and annotation data were downloaded from the Sol Genomics Network database [48]. Hmmsearch methods and BLAST searches were combined to identify *Hsf* genes in eggplant. Briefly, 325 *Hsf* gene sequences from *A. thaliana* (25), *Capsicum annuum* (25), *Carica papaya* (18), *Glycine max* (81), *M. domestica* (47), *Nicotiana tabacum* (65), *O. sativa* (36), and *Solanum lycopersicum* (26) were downloaded from PlantTFBD [56]. The downloaded Hsf proteins from different species were used as queries to search for all the possible Hsf protein sequences in the eggplant proteome file with an E-value of le-10 and identity of 60% as the thresholds. Then, Hmmsearch software was used to search for the Hsf domain (PF00447), which was downloaded from the Pfam database 32.0 [57], in the set of BLAST-identified proteins. The Hsf proteins were filtered with an E-value cutoff of 1 × 10^−5^ and at least a 60% coverage of the Pfam Hsf domain from the raw screening proteins. Furthermore, all the candidate Hsf protein sequences were analyzed to detect the DBD and coiled-coil structures using the SMART [58] and MARCOIL programs [59]. Those protein sequences, containing both a DBD and coiled-coil structure, were regarded as credible Hsf proteins. Moreover, the HEATSTER website [38] was used to confirm the 20 *SmeHsf* genes and classified them into subgroups. Finally, the Biopython module [60] was used to predict the molecular weight, isoelectric point, and other physical and chemical properties of the SmeHsf proteins. All the *SmeHsf* genes were renamed on the basis of their classifications and their phylogenetic relationships to *S. lycopersicum* and other species. 

### 4.2. Gene Structure, Domain and Motif Analyses

Gene structural information was obtained from GFF3 files and visualized using TBtools software [61]. All the full-length amino acid sequences of the SmeHsfs were used to search for conserved motifs using the MEME tool [62]. The MEME parameters were set as follows: the maximum number to be found was set to 15 and the motif window length was set 8 to 100 bp. Additionally, the conserved NLS and NES domains were predicted using cNLS Mapper software [63] and NetNES 1.1 server software, respectively. The AHA domain was identified using the conserved motif FWxxF/L, F/I/L [64].

### 4.3. Phylogenetic Analysis and Classification of SmeHsf Genes

The amino acid sequences of SmeHsf proteins identified in this study and other Hsfs from *A. thaliana*, *O. sativa*, and *S. lycopersicum* downloaded from the HEATSTER website [38] were used in the phylogenetic analysis. The complete amino acid sequences of Hsf proteins and HR-A/B domain were aligned using the MUSCLE program [65]. Subsequently, the MEGA-X program was used to construct an unrooted maximum likelihood phylogenetic tree with the Jones–Taylor–Thornton model. Additionally, a bootstrap test was replicated 500 times and a partial deletion with a site coverage cutoff of 70% was used for gap treatment.

### 4.4. cis-Element Analysis of SmeHsf Promoters

The upstream 2000 bps of *SmeHsf* genes were abstracted as the promoter sequences from the eggplant genome file. Then, the PlantCARE database was used to determine the *cis*-regulatory elements present in each gene’s promoter [41]. Besides, the upstream 2000 bps of random selected 500 eggplant genes were also used to determine the *cis*-regulatory elements using PlantCARE and compared with *SmeHsf*.

### 4.5. Plant Materials and Stress Treatments

The seeds of eggplant inbred line ‘E22’ were grown in plastic pots on the horticultural farm of the Zhejiang Academy of Agriculture Science (Hangzhou, China). At the four true-leaf stage, seedlings were moved to a growth chamber set at 16 h day (28 °C)/8 h night (24 °C) and used for experiments. For drought- and salt-stress treatments, seedlings were subjected to 100 mL of 30% PEG6000 and 300 mM NaCl, respectively, for 48 h, and for heat- and cold-stress treatments, seedlings were subjected to 38 °C and 8 °C, respectively, for 24 h. Plants were cultured under normal conditions for the control. The new leaves of five seedlings were collected as biological replicates, and each treatment had three replicates. The freshly collected samples were immediately frozen in liquid nitrogen stored at −80 °C for RNA isolation.

### 4.6. RNA Extraction and qRT-PCR Analysis

TRIzol reagent (Invitrogen, Carlsbad, CA, USA) was used to independently extract total RNAs of all the samples, and genomic DNA contamination was removed using DNase I. Then, RNA concentrations were measured using a NanoDrop2000 microvolume spectrophotometer (Thermo Fisher Scientific, Wilmington, DE, USA), and the RNA integrity was checked by 1.5% agarose gel electrophoresis. PrimeScript RTase (TaKaRa Biotechnology, Dalian, China) was used for first-strand cDNA synthesis following the manufacturer’s instructions. The primers for qRT-PCR reactions were designed using Primer Premier 5.0, and the *SmEF1a* gene was used as a stable reference gene [66]. The qRT-PCR reactions were performed on a TIB8600 machine using AceQ^®^ qPCR SYBR^®^ Green Master Mix kits (Vazyme Biotechnology, Nanjing, China) with the following settings: 95 °C for 5 min; followed by 40 cycles of 95 °C for 15 s and 60 °C for 30 s. The relative expression levels of *SmeHsf* genes were calculated using the 2^−ΔΔCt^ method [67]. The analysis included three biological replicates for each sample. All the primer sequences are listed in Table S4.

### 4.7. Statistical Analyses

The statistical analysis was carried out by calculating the average values and standard errors for the three replicates. SPSS software version 16.0 was used to determine the significant differences between controls and stress treatments using a one-way ANOVA procedure and post hoc analysis. A *p* value ≤ 0.05 indicates a significant difference and is represented by an asterisk (*); a *p* value ≤ 0.01 indicates a very significant difference and is represented by two asterisks (**).

## 5. Conclusions

In the present study, 20 full-length *SmeHsf* genes were identified in the eggplant genome. These *SmeHsf*s were comprehensively characterized using a systematic approach comprising analyses of sequence characteristics, phylogeny, classifications, gene structures, and motif compositions. Moreover, a qRT-PCR analysis of *SmeHsf* expression levels in response to various abiotic stresses indicated that *SmeHsf*s not only play crucial roles in heat tolerance, but also increase the tolerance levels to various abiotic stresses. This comprehensive analysis provides candidate genes for future functional analyses under stress conditions and also lays the foundation for investigating molecular mechanisms of abiotic stress tolerance in plants.

## Figures and Tables

**Table 1:** Physicochemical characteristics and classification of Hsf genes in eggplant.

Number	Gene Name	Gene Code	Subgroup	Protein Length (aa)	Molecular Weight (kDa)	Aromaticity	Instability Index	Isoelectric Point	GRAVY ^1^
1	SmeHsfA1b	Sme2.5_02334.1_g00004.1	A1	496	54.84	0.05	52.58	5.14	−0.48
2	SmeHsfA1e	Sme2.5_00204.1_g00007.1	A1	478	53.81	0.06	51.96	5.88	−0.61
3	SmeHsfA3	Sme2.5_00292.1_g00007.1	A3	494	55.08	0.10	53.86	4.60	−0.58
4	SmeHsfA4a	Sme2.5_01013.1_g00005.1	A4	403	46.04	0.07	42.59	5.15	−0.78
5	SmeHsfA4b	Sme2.5_01314.1_g00005.1	A4	421	48.36	0.09	54.15	5.35	−0.74
6	SmeHsfA4c	Sme2.5_04312.1_g00009.1	A4	377	43.01	0.07	47.45	5.14	−0.76
7	SmeHsfA5	Sme2.5_09846.1_g00002.1	A5	475	53.31	0.07	57.57	5.51	−0.78
8	SmeHsfA6a	Sme2.5_00065.1_g00020.1	A6	357	41.70	0.10	45.43	5.15	−0.93
9	SmeHsfA6b	Sme2.5_08000.1_g00008.1	A6	324	37.93	0.08	47.19	5.50	−0.83
10	SmeHsfA6c	Sme2.5_04149.1_g00004.1	A6	343	39.84	0.09	59.73	5.99	−0.79
11	SmeHsfA8	Sme2.5_08951.1_g00003.1	A8	374	43.27	0.10	58.33	4.72	−0.62
12	SmeHsfA9a	Sme2.5_00023.1_g00025.1	A9	410	47.37	0.09	50.08	5.29	−0.55
13	SmeHsfA9b	Sme2.5_03412.1_g00012.1	A9	317	36.77	0.12	41.31	9.00	−0.74
14	SmeHsfA9c	Sme2.5_04312.1_g00005.1	A9	340	38.48	0.07	48.87	5.93	−0.86
15	SmeHsfB1	Sme2.5_00010.1_g00004.1	B1	481	53.57	0.07	30.35	5.24	−0.67
16	SmeHsfB2a	Sme2.5_13301.1_g00001.1	B2	341	38.30	0.07	60.33	6.35	−0.62
17	SmeHsfB2b	Sme2.5_02712.1_g00007.1	B2	331	36.21	0.06	55.45	5.18	−0.44
18	SmeHsfB3a	Sme2.5_00159.1_g00006.1	B3	213	24.62	0.08	52.04	9.44	−0.79
19	SmeHsfB4a	Sme2.5_01029.1_g00008.1	B4	357	40.64	0.09	60.84	7.73	−0.60
20	SmeHsfC1	Sme2.5_04829.1_g00004.1	C1	352	39.42	0.09	68.53	6.14	−0.65

^1^ GRAVY—The abbreviation for grand average of hydropathy values.

**Table 2:** The function domains and their position in SmeHsfs.

Gene	DBD	HR-A/B	NLS	NES	AHA
SmeHsfA1b	13–106	143–196	(205) NNSKKRRLLVSNY	(150) ILM	Na
SmeHsfA1e	10–103	140–193	(211) ITGMNKKRRFP	Na	Na
SmeHsfA3	78–171	196–249	Na	Na	Na
SmeHsfA4a	10–103	136–189	Na	Na	(341) DVFWEQFLTE
SmeHsfA4b	16–109	139–192	Na	(236) LEM	(245) INFWERFLYG; (354) DVFWQQFLTE
SmeHsfA4c	10–104	136–189	(203) NDRKRRFPG	Na	(343) DVFWEQFLTE
SmeHsfA5	13–106	133–186	(202) ISAFSKKRRLP	(193) LAQKLESMDI	(422) DVFWEQFLTE
SmeHsfA6a	34–127	159–212	(220) EIRNKRKRQID	Na	(314) EGFWEDLLNE
SmeHsfA6b	29–122	153–206	(115) LLRTIKRRKTTNF; (226) EINKKRRRPID	(265) VALNM	Na
SmeHsfA6c	33–126	160–213	(119) LLRNIKRRKTP; (222) QQKGKRKEIEEDITKKRRQPI	(191) LRL	(305) MGFWEELFND
SmeHsfA8	10–103	138–191	Na	Na	Na
SmeHsfA9a	46–139	164–217	(134) INIKRRKQYP; (229) KQGKKRKLCDAQF	Na	Na
SmeHsfA9b	29–122	147–200	Na	Na	Na
SmeHsfA9c	36–129	160–213	(219) DTRKRPCLV	Na	(269) REFWEKLFED
SmeHsfB1	6–99	161–193	(254) KEKKKKRGPD	Na	Na
SmeHsfB2a	22–115	177–209	Na	Na	Na
SmeHsfB2b	21–114	196–228	Na	Na	Na
SmeHsfB3a	2–84	137–169	(190) EMERKRKRVEL	Na	Na
SmeHsfB4a	21–114	201–233	(203) NERKRRLPG	(342) LEKNDLGL	Na
SmeHsfC1	1–83	108–147	(169) REKKRRLMIS	(155) LMEKERSKRLSL	Na

DND-binding domain (DBD), oligomerization domain (HR-A/B), nuclear export signal (NES), nuclear localization signal (NLS), activator motifs (AHA). Numbers in brackets reveals the position of the first amino acid of NLS, NES, and AHA domains in the sequence; Na—no domains detected.

## Supplementary Materials

Supplementary Materials can be found at https://www.mdpi.com/2223-7747/9/7/915/s1. Table S1: The alignment information and scaffold position of the SmeHsfs, Table S2: The protein sequences of the SmeHsfs, Table S3: cis-Element analysis of SmeHsf promoters, Table S4: qRT-PCR primers for the SmeHsf genes.
